# A Qualitative Investigation of Factors Influencing the Dietary Intakes of Professional Australian Football Players

**DOI:** 10.3390/ijerph18084205

**Published:** 2021-04-15

**Authors:** Sarah Jenner, Regina Belski, Brooke Devlin, Aaron Coutts, Thomas Kempton, Adrienne Forsyth

**Affiliations:** 1 School of Allied Health, Human Services and Sport, La Trobe University, Bundoora, VIC 3086, Australia; b.devlin@latrobe.edu.au (B.D.); a.forsyth@latrobe.edu.au (A.F.); 2 Carlton Football Club, Carlton, VIC 3054, Australia; tom.kempton@carltonfc.com.au; 3 School of Health Sciences, Swinburne University of Technology, Hawthorn, VIC 3122, Australia; rbelski@swin.edu.au; 4 Human Performance Research Centre, University of Technology Sydney, Moore Park, NSW 2007, Australia; Aaron.Coutts@uts.edu.au

**Keywords:** carbohydrate, body composition, Australian football, qualitative, education

## Abstract

(1) Background: Many professional Australian Football (AF) players do not meet recommended sports nutrition guidelines despite having access to nutrition advice. There are a range of factors that can influence players′ ability to meet their nutrition goals and awareness of the barriers players face is essential to ensure that dietary advice translates into practice. Therefore, this qualitative research study aimed to explore the factors influencing AF players’ dietary intakes and food choice. (2) Methods: Semi-structured interviews were conducted with twelve professional male AF players. (3) Results: Less experienced players restricted their carbohydrate intake to meet body composition goals, particularly during preseason and surrounding body composition assessment. During the competition season players had a greater focus on performance and placed more emphasis on carbohydrate intake in the lead up to matches. Players felt nutrition goals were easier to achieve when dietary choices were supported by their families and peers. One-on-one consultations provided by a sports dietitian were players′ preferred mode of nutrition intervention. Individualized nutrition advice is required for less experienced AF players who may be vulnerable to unsustainable dietary habits. Experienced AF players can support junior teammates by promoting positive team culture related to body composition, nutrition and performance.

## 1. Introduction

Personal food choices are complex and influenced by the interaction between an individual′s sense of self and daily food activities [1]. A variety of factors such as knowledge, attitude and sociodemographic characteristics challenge personal reflection and can influence an individual’s view on eating practices [1]. Eating in a social setting with family, friends and partners has also been linked to food choice. These social settings may influence how individuals perceive how they are judged by others, influencing their food choice, body image and self-esteem [1,2]. Birkenhead and Slater (2015) highlighted a range of factors that can influence individual food choice for players including taste preferences, cultural and religious beliefs, access to food, price, and convenience. Involvement in professional competition and awareness of nutrition for performance and recovery are thought to be further drivers of good nutrition [3]. Additionally, potential concerns regarding body composition, stage of training and performance expectations have been suggested more recently as drivers of food choice for athletes, yet research on factors influencing food choice in athletes is largely unexplored [3].

For athletic performance, a well-designed diet that meets energy and macronutrient needs, as well as proper timing and distribution, is essential [4,5,6,7]. Despite the presence of specific nutrition recommendations within team sport environments, research over the past decade has found many players do not meet these recommendations for energy and/or carbohydrate intake [8,9]. More recently, concerns have been raised regarding the influence of body composition goals on players′ dietary intake and perception of their nutrition goals [3,4]. Attempts to achieve optimal physique and meet body weight goals for performance can increase the presence of unrealistic weight loss practices, where in extreme cases elimination of one or more food groups or the consumption of restrictive dietary plans with low macronutrient density are evident [4]. Pressure from peers or culture within a sport to meet performance goals can also contribute to unsustainable dietary practices [3].

Australian Football (AF) is a unique sport that encompasses a range of physical demands including aerobic fitness, speed, power and agility [10]. The AF season is divided into preseason (~3 months) and competition season (~6 months), which are performed over the summer and winter months, respectively [10]. AF players′ nutrition demands will be related to the training and competition undertaken. Earlier studies in AF have reported that AF players′ dietary intakes do not align with sports nutrition recommendations for carbohydrates and that they have poor nutrition knowledge (NK) [8]. In particular, previous research undertaken by the current research team assessed dietary intakes of the same cohort of Australian Football players and found using self-reported food records that, on average, no athlete included in research met sports nutrition recommendations for energy and carbohydrates [11]. In addition, athletes′ nutrition knowledge was assessed using a validated tool (i.e., Nutrition for Sport Knowledge Questionnaire) and showed that athletes had poor NK (i.e., 46% mean score) [11,12,13]. Researchers also assessed the association between AF player′s dietary intake and education status, level of playing experience and NK, finding only significant associations between education status and vegetable intake, intake of energy and NK scores, and energy intake, protein, calcium and fibre [11].

Access to nutrition education and advice is important to improve NK and support dietary intakes to meet nutrition needs related to performance and recovery. Nutrition education for players has been shown to improve NK [12]. Understanding the factors that influence players′ food choice may help sports dietitians to individualize nutrition intervention to players′ needs [3]. Therefore, the present study aimed to qualitatively explore the factors influencing dietary habits and food choices of AF players.

## 2. Materials and Methods

A qualitative approach using semi-structured interviews with AF players was selected to elicit players′ views on factors influencing their dietary habits and food choice [14]. AF players were approached and recruited in person by one researcher (SJ), from one club competing professionally in the Australian Football League (AFL). All players were selected using purposeful sampling to ensure the sample included players over the age of 18 years and with a variety of AFL experience levels (range: 2–12 years). 

At the time of data collection, athletes had access to one-on-one consultations with a sports dietitian; however, due to limited hours of employment of the sports dietitian, additional nutrition education was provided to athletes via group education, including cooking classes. In-house catering was not available, however athletes had access to external catering options including local restaurants and an in-house café. To monitor body composition, Dual-energy Xray Absorptiometry (DXA) scans were undertaken four times across the AFL season, with preparation guidelines provided to athletes that align with best practice protocols for athlete presentation, placement on scanning bed and technician precision [11].

Interviews took place during the competition season. To protect player privacy, the date of data collection is not reported. Interviews were conducted within the club′s facilities and were audio-recorded with players′ consent. Each interview ran for approximately 30 min and was facilitated by a researcher (SJ) who also served as the club sports dietitian. This was beneficial for data collection, given that the researcher had established rapport with the recruited players and understood the demands and player experiences within the specific team sport environment.

Questions were constructed using inquiry logic, were open-ended and allowed players to draw on their own nutrition goals and experiences (Table 1). Interviews were composed of three main sections. Section one aimed to explore factors that influence (including those that support or hinder) dietary intake and food choice. Based on the factors highlighted by Birkenhead and Slater (2015) players were probed to consider factors such as taste, access, teammates and training and whether these influenced their dietary behaviors [3]. Section two explored players′ preparation including dietary patterns in the weeks and days leading to a body composition assessment (DXA scan) period. Jenner et al. had previously hypothesized that AF players adjusted dietary intake leading up to a DXA scan [11]. Therefore, to explore this theory, section two included questions designed to provide insight into AF players′ dietary patterns in the weeks and days surrounding body composition assessment periods. Lastly, section three considered players′ preferred method of nutrition education and types of nutrition intervention that were most helpful in guiding food choice. This section allowed players to provide feedback regarding the utility of the sports dietitian and their own experiences with nutrition intervention.

All interviews were audio-recorded and manually transcribed in full by one researcher (SJ). All participants were asked to review and confirm the transcript of their interview. Using methods described by Braun and Clarke, a thematic analysis was undertaken by two members of the research team (SJ and AF) [15]. The researchers independently reviewed each transcript and generated initial codes. An inductive approach was used in the analysis of sections one and three, while a deductive approach was used for section two where questions were designed to explore a pre-existing theory. Using a cross examination approach codes were used to identify emerging categories and sub-categories and through discussion themes were established [15]. Initial codes were used to group data into themes and to identify supporting quotations [15].

All study activities were approved by the La Trobe University Human Ethics Committee (S17-025).

## 3. Results

### 3.1. Participants

Thirteen AF players were approached to participate in the study, with twelve players providing informed written consent to participate in semi-structured interviews. Mean age and AFL playing experience of players included was 23.0 ± 4.3 years and 5.5 ± 4.1 years, respectively. Mean weight (kg) and height (cm) and body fat percentage of players (% BF) included was 86.7 ± 7.4 kg, 187.6 ± 7.6 cm and 11.3 ± 2.3 % BF, respectively. Highest education level obtained by players included completed school/high school (33.3%), completed/enrolled in TAFE/diploma (33.3%) and completed/enrolled in university course (33.3%).

### 3.2. Categories, Sub-Categories and Themes

**Figure 1:**
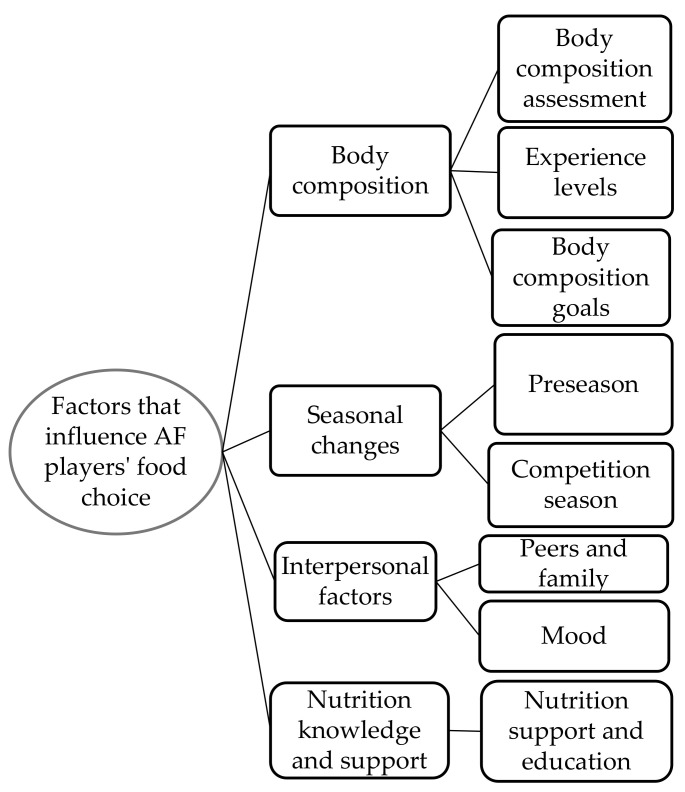
Factors that influence Australian football players’ personal food choice.

Researchers developed four categories that helped to explore factors behind AF players’ food choice and dietary intake behaviors. Players highlighted a range of factors that influenced their food choice. Categories to describe factors that influence AF players′ food choice include (1) body composition, (2) interpersonal factors (i.e., peers, family, access), (3) seasonal changes and (4) nutrition knowledge and support (Table 2). The influence of body composition and interpersonal factors were highlighted as the main categories to explain decisions behind food choice during an AFL season. Dietary strategies such as the manipulation of carbohydrate intake and excessive fluid intake surrounding times when body composition assessments were being undertaken were reported by players. A thematic map (Figure 1) was created to demonstrate the influence of these four key categories on food choice and their relationship with dietary behaviors.

### 3.3. Category One: Body Composition

#### 3.3.1. Perceived Pressures Associated with Body Composition Assessment and Goals

Body composition goals were developed by sports professionals for each AF player. Players reported that a range of individuals were included in the decision-making behind body composition goals including strength and conditioning coach, sports dietitian, sports scientist, coach and/or themselves. Many players identified their own body composition goals, based on the physique of other players of the same body type or those competing in the same playing position.

Goals developed by the players themselves were often unrealistic and unnecessarily low in body fat. Standards related to body composition were driven by teammates and staff (previously outlined) and were perceived as a source of pressure by players.

*“I don′t think there′s enough attention to how much muscle you′ve gained or lost’…there′s a lot made out if you have put on a kilo of fat.”*

*“And if you don’t come back better than last time yeah there′s a few pressures…”*

#### 3.3.2. Dietary Strategies Used to Meet Body Composition Goals

Players provided insight into their usual dietary behaviors surrounding a DXA scan. Players reported changes to dietary intake including reductions in carbohydrate intake and excessive fluid intake two weeks before a designated DXA scan. Furthermore, players reported adjusting or removing carbohydrate-rich foods from their diets in the week leading into an assessment period. They described a constant compromise between consuming enough carbohydrate for energy during training and ensuring intake supported body composition goals.

*“…generally, in the weeks leading up, one to two weeks before a scan I cut out carbs a lot. Basically, almost that I am not eating carbohydrates at all because I do burn a lot of fat when I am doing that.”*

*“I would have shredded right up to the scan and drank a couple of liters of water right before, but I think I have become more relaxed in the last year.”*

Players′ dietary habits were heavily influenced by the presence of body composition goals, with most of the changes made to habitual dietary intake undertaken during periods where body composition was being assessed. Pressures experienced by players to meet body composition goals were reported, with many acknowledging that dietary intake during these times did not represent usual intake. Players identified that these intakes may not be beneficial for their performance, recovery, and health.

*“…Because sometimes you can stress and the patterns you do throughout the year and patterns before a DXA can be unhealthy.”*

#### 3.3.3. Experienced Players Felt Less Pressure to Meet Body Composition Goals

Performance and training needs were the main drivers of food choice for experienced AF players. These players experienced less pressure surrounding body composition and used words such as ‘strong’ and ‘powerful’ when discussing their body composition goals. Experienced players provided insight into the presence of body composition goals within their team environment and the influence meeting these goals have had on their dietary patterns and performance in the past.

*“Maybe a few years back it was about getting as low as you can be, and I don′t think that′s the best way to go about it.”*

*“…I was 10.4% [body fat] once and it′s such a fine line because I got to 10.4% in preseason, and I was so light and didn′t have power in the contest but wasn′t getting applauded for how I was running.”*

Experienced players identified a growing culture of support and understanding for their younger teammates, who experience greater levels of stress surrounding body composition assessment periods. Players spoke of the importance of performance on the field and further highlighted the importance of not removing carbohydrate-rich foods in order to meet their training and performance needs.

*“…I think it all needs to be around performance and performance in training. You can′t be eating less carbs and feeling flat at training.”*

Players reported difficulties in meeting body composition goals and dietary strategies used to alter body composition to reach these goals. Experienced players felt less pressure to meet body composition goals in comparison to young AF players. This suggests that more experienced players have a greater understanding of their body type, performance and diet and the impact body composition changes have on their performance. Additionally, younger players were more vulnerable to unsustainable dietary practices, with many developing their own body composition goals without expert advice.

### 3.4. Category Two: Interpersonal Factors

Players provided insight into the interpersonal drivers behind food choice, many reporting social influences and relationships (i.e., family and peers) as the main influences on food choice. Furthermore, players felt dietary intake behaviors were influenced by changes in mood. 

#### 3.4.1. The Influence of Peers and Family

When players felt supported by their family and peers (including teammates) they were more likely to demonstrate positive dietary behaviors associated with performance and recovery. Family members and partners were the main influences on players′ food choice. Players expressed that they were more likely to choose foods that did not align with their nutrition and performance goals if their social networks were not conducive to healthy eating.

*“You come home, and your partner is eating burgers and chips and then you have to go and choose healthy things off the menu, it isn′t going to work that well. It doesn′t affect me as much because she (my partner) eats well.”*

Players identified teammates as influences on their nutrition noting that team culture had a significant influence on their food choice, particularly around training sessions. Players that did not meet their body composition goals or did not choose foods related to their health and performance needs were viewed as unprofessional by their peers. Players experienced frustration when teammates failed to meet their specific body composition goal.

*“Yeah, I think that′s the standard and what everyone expects and if you are not (eating to support your goals) then you are not seen as professional.”*

Players who struggled to reach their nutrition goals often found support from their teammates to meet these goals. Those who engaged in positive dietary behaviors, such as cooking within the home and meal preparation, felt these behaviors were influenced by the professionalism demonstrated by others. Players also reported that teammates were key sources of nutrition information and advice regarding optimal diet and dietary strategies.

*“We have a fair few other players that also bring their lunch in. So, we hang out around the café, eat our food and have a cup of coffee up there.”*

*“I lived with another player who was elite with his meal preparation. So, I think I learnt by living with him; whatever he was eating I was eating, and I learnt that way.”*

#### 3.4.2. The Influence of Mood on Food Choice

Variations in mood appeared to influence players′ food choice, with positive mood linked with greater motivation to meet nutrition goals and positive food behaviors. However, those that experienced low mood levels reported a lack of motivation to choose positive food behaviors.

*“…probably a big one is mood. Depends on the mood, if you are in a bad mood, I′ll tend to eat bad food, if I′m in a good mood I′ll probably stick to routine.”*

Peers, family, and changes to mood were found to contribute to players′ food choice and influence players′ dietary behaviors. Taken together, players reported positive dietary behaviors and intakes when supported by their families and peers. Furthermore, changes in mood influenced players′ food choice, with decreased mood associated with more discretionary food choices.

### 3.5. Category Three: Stage of Competition Season (Preseason and Competition Season)

Differences in training loads and nutrition motivations between the preseason and in-season were reported as influences on players′ dietary intakes. During the preseason, limiting factors such as appetite suppression post-training and limited time to eat on main training days were experienced by players. Additionally, during preseason, players perceived that there was a greater focus on meeting body composition goals. In comparison, during the competition season players reported a greater focus on meeting nutrition needs for carbohydrates, to ensure their match day performance and recovery needs were met.

#### 3.5.1. Preseason Influenced Dietary Intake (i.e., Reduced Carbohydrate Intake)

During the preseason, players reported a variety of barriers to meeting nutrition goals with many reporting that greater training loads and intensities influenced appetite regulation post-training. Appetite suppression was experienced post-training and identified as a barrier to recovery nutrition. Players reported difficulties meeting nutrition goals when training intensities and energy expenditures were high. Furthermore, difficulties with appetite (i.e., suppressed appetite) were also experienced when temperatures during preseason were warmer than usual. However, the overall volume of food consumed was reported as greater during the preseason period. Players reflected that most of their dietary intake was consumed in the hours after training or on recovery days.

*“Not straight after training. But when I′m in the car driving home because I have an hour′s drive that′s when I get hungry.”*

Limited time to eat during training days due to scheduled meetings and other commitments influenced players′ recovery nutrition post exercise. Players reported that nutrition post-training was not a priority and that time made available to eat was limited.

*“Yeah, preseason training, you are usually off to one thing and the next, sneak the food in when you can.”*

As noted earlier, body composition goals were also reported as a major focus during preseason with players reporting these goals as driven by staff and teammates. Players reported that without the presence of match play and competition, body composition was used as a driver for their training and dietary intake.

*“Performance in preseason is like how you are physically but with games or in-season it′s performance in games… So, you′re judged on how you are looking and how you present in preseason and in-season it′s how you play.”*

Whilst players reported pressures associated with meeting body composition goals, some reported that having a focus on their nutrition and body during preseason allowed them to maintain focus.

*“In preseason it′s nice to have a focus on your body and your nutrition as well because there aren′t games to focus on so that′s how you are performing, and I like that in preseason.”*

#### 3.5.2. Competition Season Influenced Dietary Intake (i.e., Increased Carbohydrate Intake)

The main dietary influences during the competition season were team selection and performance pressures, and the effects of high-intensity activity during games on appetite. Players reported that, during the competition season, food choice was mostly made to complement competition performance and recovery needs. Players reported strategies such as carbohydrate periodization and loading and monitoring of hydration to prepare for competition. Players reported that dietary intake did not change surrounding body composition assessments during this time, with many reporting a greater consumption of carbohydrate-rich foods during these periods.

*“Depending on when we are training that’s how I alter my carbohydrate intake…I eat more carbs in-season (competition season), just because I am more conscious of performance and recovery.”*

*“What fuel I need for game day, mainly around the carbs in-season. I think that′s the main thing that changes are the carb periodization thing that the dietitian introduced and trying to get that right more often than not. That′s the main reasoning why I eat the food I eat.”*

However, for those players needing to alter body composition during competition season, these goals were likely to impact their preparation and overall competition performance.

*“I play better when I am relaxed, and not thinking about that kind of stuff (body composition).”*

On competition days, players reported that feelings of nervousness and reduced appetite limited their dietary intake leading into a match.

*“I am hungry in the morning before training sessions, games are different. I don′t really eat all that much.”*

Recovery nutrition post-match was also influenced by a lack of appetite.

*“Yes definitely, initially I have a lack of appetite after a match. It will take me two–three hours to get something back in.”*

Food choice and dietary habits seemed to be driven by stage of competition season, with more unsustainable dietary practices demonstrated during the preseason period. During competition season, players appeared to make food choices related to their performance and recovery needs, although match-day presented challenges around food intake.

### 3.6. Category Four: Nutrition Knowledge and Support

#### Nutrition Education and Support Preferred by AF Players

Players were asked about their preferred methods of nutrition guidance. Individualized nutritional advice, which includes one-on-one consultations, was preferred in comparison to the provision of nutritional information via group education. Players felt best supported when nutritional advice was individualized to their needs, lifestyle and skill level, for example, cooking classes, and sessions on label reading for those with lower levels of prior knowledge about food and nutrition.

*“One on one is good for me. In terms of learning the basics in a group is fine. But if you are trying to personalize training loads, diets, and intakes, one on one is by far the best because everyone is different.”*

Experienced players acknowledged that nutrition advice in the past had been provided from a range of information sources. However, players felt sports dietitians in more recent times had a greater role in the provision of nutrition advice within the team sports environments.

*“…Other staff would be all about body fat percentage. Where the dietitian, is more levelled with it all. Like getting enough carbs in and having a balanced diet, in comparison to always body fat percentage.”*

Players felt they had good nutrition knowledge; however, they acknowledged that ongoing support and access to nutrition resources were important for their motivation. Furthermore, players felt greater support to keep on track and meet their nutrition goals when a sports dietitian was present within their sporting environment.

*“If I have specific questions, I like coming and asking [the dietitian] and that gets the convo going and it′s more individually tailored.”*

Access to a sports dietitian may be a protective factor for the impact of body composition on dietary habits, especially for players that experience performance-related pressures. Those that felt supported to meet their nutrition goals appeared to make food related decisions based on their health and performance.

## 4. Discussion

This study aimed to qualitatively explore factors influencing dietary habits and food choice of AF players. Players reported a range of factors that influence food choice and their ability to meet their nutrition goals. The main influences included pressures to meet body composition goals and the role of peers and family (including partners). Individualized advice provided by a sports nutrition professional (i.e. sports dietitian) was perceived as the best source of nutrition information and support. 

### 4.1. Feelings and Pressures Associated with Body Composition Goals

Pressures associated with meeting body composition goals were highlighted as a major influencing factor on AF players′ nutrition choices. This finding is supported by previous qualitative research by Heaney and O’Connor [16] which reported that body composition was also perceived as a barrier. In their study interviews were undertaken with Australian athletes, coaches and sports dietitians from a diverse range of sports and revealed that meeting body composition goals (i.e., physique) was a barrier to maintaining good nutrition [16]. They also found that coaches were more concerned about body composition than players and sports dietitians [16]. Interestingly, in the current study, experienced AF players were less likely to be influenced by the presence of body composition goals, reporting match day performances and recovery as their main drivers for food choices and meeting their nutrition goals. Although previous research has not found significant differences in carbohydrate and energy intakes between levels of playing experience [11,17], Bilsborough et al. (2016) found that inexperienced AF players had significantly greater dietary fat intakes [17]. Taken together, our findings suggest that younger or inexperienced players may be more vulnerable to unsustainable nutrition practices. This draws attention to the potential benefit of including experienced players in nutrition messaging and promoting a positive culture around food and performance. Furthermore, due to the presence of pressures related to body composition, nutrition education that specifically focuses on sustainable nutrition practices for body composition changes may be beneficial for those involved in the monitoring and development of body composition goals (i.e., high-performance teams and coaches). Success of nutrition education programs with sports specific staff has been explored in a study by Jacob et al. (2016) who found that a theory-based nutrition intervention helped coaches to provide more accurate recommendations on sports nutrition [18]. Furthermore, the inclusion of standardized protocols prior to body composition assessment may help to minimize error and promote sustainable nutrition practices leading up to a DXA scan [19].

### 4.2. Dietary Practices Associated with Adjusting or Maintaining Body Composition

Maintaining optimal body composition, in particular the building and maintaining of muscle mass throughout a competitive season, is important to reduce the risk of injury and illness and enhance performance [4]. There is, however, limited research to quantify recommended body composition goals for AF players [11]. What is known is the importance of muscle mass for strength and power, which are important characteristics to meet the physical demands of AF [10,20]. The use of body composition assessment methods, such as DXA, may be warranted to provide professional players with feedback on energy balance and changes to muscle mass [21]. In this study, AF players reported adjusting carbohydrate intake and/or removing carbohydrate foods from their diet in the lead up to a body composition assessment, which may stem from misconceptions regarding the role of carbohydrates for sports performance. Many players associated high carbohydrate intakes with increased fat mass. The challenges of changing perceptions and misconceptions with regards to the amount of carbohydrate required for team sports players has been highlighted in past research [8,22,23]. International carbohydrate recommendations have evolved in recent times, with greater focus on carbohydrate periodization strategies that focus on the timing and manipulation of carbohydrate intake [23]. Manipulating dietary carbohydrate intake to align with training outputs can be a sustainable practice to adjust body composition and enhance training adaptions [22]. Furthermore, it may be unnecessary for AF players to consume high carbohydrate intakes (i.e., >8 g·kg^−1^·day^−1^) for consecutive training sessions without adjustments made for training outputs [22,24]. In the current study, those players who adopted strategies of carbohydrate periodization reported more sustainable dietary intake habits surrounding body composition assessment periods. To support sustainable dietary intakes, AF players may benefit from greater individualized advice with a focus on tailoring carbohydrate intake to training demands. Future research into the development of realistic body composition goals for AF players may be beneficial for staff working with these players. Whilst realistic body composition goals are important for player wellbeing, goals should not overshadow the importance of consistent athlete monitoring and an individualized approach to nutrition.

### 4.3. Factors That Influence Dietary Intake of AF Players (i.e., Barriers to Meeting Nutrition Goals, Peers and Family, Preseason versus Competition Season Changes)

The social act of eating and the surrounding environment may be as important as the advice and education provided to players to improve nutrition knowledge and support food choice [25]. Peers and family (including teammates and partners) were perceived by players as one of the main influences on their food choice. Similarly, Trakman et al. (2019) found that 50% of Australian athletes (*n* = 410) from a range of individual and team sports received nutrition information from family and friends, coaches and teammates [26]. Our findings suggest that families and teammates are sources of motivation for food choice, with many AF players reporting a greater ability to meet nutrition goals when supported by their teammates and families. An athlete′s social network outside of the team environment can additionally support or hinder nutrition messages, particularly if players do not have the necessary knowledge and strategies to make informed food choices. To better support AF players′ dietary intakes outside of the team environment, future research should explore the effectiveness of nutrition education programs developed for families and peers on players’ ability to meet their nutrition goals. For vulnerable groups such as young or inexperienced players, building peer support around diet is essential and may be promoted by the inclusion of experienced players in nutrition messaging and education programs, with guidance from sports nutrition professionals.

The influence of seasonal changes on AF players′ dietary intakes and body composition has been explored in previous research [17,27]. Typically, during the preseason training phase, decreases in fat mass and increases in muscle mass are observed. However, during the competition phase, the challenge is to maintain muscle mass and mitigate against excessive increases in fat mass [17]. Bilsborough et al. (2016) assessed the dietary intakes of AF players over a season and found significantly greater dietary fat intakes at the start of preseason, with no differences in energy or carbohydrate intake throughout the preseason and competition phases [17]. During preseason there may be a greater focus on body composition, due to the absence of competition (i.e., match-day) and therefore limited available measures to assess performance. Furthermore, preseason will be an important period for players to prepare physically for competition success. Bradley et al. found that professional rugby union players had greater intakes of protein in preseason which exceeded recommendations (2.5–2.6 g·kg^−1^·day^−1^) as a result of players practicing carbohydrate periodization to optimize body composition [24]. To avoid unwanted energy deficits that may arise from practices associated with adjusting body composition (i.e., manipulating carbohydrate intake), a greater focus on individualized nutrition and tailoring intakes to training loads during preseason for this athletic population is recommended.

### 4.4. Sports Dietitians in the Team Sport Environment and the Importance of Having Individualized Nutrition Advice

Sports dietitians work to motivate and guide players to make food choices that align with performance and recovery demands. Whilst the capacity for sports dietitians in team sport environments may be limited due to economic and time constraints [16], many players report having access to advice and information from a sports dietitian or nutritionist to some degree [26,28,29,30]. Due to these constraints, an understanding of the nutrition intervention desired by players and teams can be beneficial to advocate for nutrition intervention in team sport environments. In this study, AF players favored working one on one with a sports dietitian, in comparison to the provision of nutrition intervention via group education (including cooking programs) and other information sources (i.e., websites, online resources). The benefits of tailored advice to support players′ nutrition goals has been established, with significant changes to players′ dietary intakes, with increases in carbohydrate and protein intake and NK, observed after completion of an individualized nutrition counselling program [31]. Furthermore, positive associations have been observed between dietary intake and access to a sports dietitian, with Hull et al. reporting significant differences between dietary intake of players who worked with a sports dietitian compared to those who did not [32,33]. Players who reported receiving regular advice from a sports dietitian had reduced fast food intake, more regular pre-training breakfast intake and prepared more meals at home [33]. Taken together these findings suggest that providing access to a sports dietitian may improve adherence to sustainable nutrition practices; however, more research regarding efficacy of education methods to improve AF players’ dietary intakes is required.

### 4.5. Limitations

The nature of the cohort included in this study (i.e., small sample size, *n* = 12 and recruited from one club) may limit the generalizability of the results. However, a redundancy in information obtained was observed within the 12 interviews, suggesting data saturation was achieved and no further interviews were required. This study only included male AF players, so more research is required to explore female AF players′ perceptions of nutrition, body composition and type of nutrition intervention preferred. With only one researcher (SJ) conducting interviews with players, interview bias was highlighted as a potential confounding factor when developing the study design. It was potentially beneficial that the interviewer was connected directly with the players in the club environment, improving the candor of the responses due to previously established relationships. However, due to the interviewer’s affiliation with the club, it may have also limited players′ responses including voicing concerns and providing feedback. The ‘Pygmalion effect’ described by Rosenthal and Jacobson is a theory used to describe the phenomena whereby expectations of an individual may influence the responses collected, therefore, with regards to our research, responses may have been influenced by the presence of the team sports dietitian who collected interview responses [34]. To reduce the risk of confirmation bias, a separate researcher (AF) was included in the analysis of data collected and the development of categories and themes. Despite these limitations, this study provides the first insight into professional AF players′ perceptions of factors influencing food choice and provides feedback on the presence of a sports dietitian in a professional team sport environment.

## 5. Conclusions

Whilst many AF players understand the importance of good nutrition practices, the pressure of meeting body composition goals and the influence of peers and family were acknowledged as influences on their dietary intake and ability to meet nutrition goals. During periods where body composition was being assessed, players reported their carbohydrate intake decreased in the weeks, and fluid intake increased in the days leading into a DXA scan. Younger or inexperienced AF players were found to be more likely to report changes in dietary intakes surrounding body composition assessment periods and therefore may require additional support to ensure food choice meets performance and recovery goals. To further promote messages related to healthy eating behaviors and positive culture surrounding body composition, sports dietitians may aim to include experienced players as a support tool in nutrition education programs. Nutrition education regarding the importance of periodized carbohydrate intake for performance and the impact of body composition pressures on players may also be beneficial for support staff involved in the development of body composition goals.

## 6. Future Directions for Research

More research is required to explore the efficacy of nutrition interventions and types of education to improve dietary intake and inform appropriate food choice within the team sport environment where access to individualized nutrition advice is limited. There is also a need to expand on this work with a wider cohort of players drawn from other AF clubs including female AF players to confirm that the barriers to players meeting nutrition goals observed in this playing group are applicable across the competition. Furthermore, exploring the factors that influence food choice in a range of individual and team sport environments would allow sports professionals, including sports dietitians, to better understand their athletes′ needs and tailor recommendations accordingly.

## Figures and Tables

**Table 1:** Semi-structured interview questions.

Category Explored	Question	Logic
Section one Influences on food choice and dietary habits	What are the key factors you think influence your food choices (Prompts: Taste, access, teammates, training)? Does your food intake vary over the season? Is your food intake different in preseason versus in-season? Do training sessions (e.g., intensity, limited time) impact on your food choices or dietary habits?	Establish internal and external factors that influence food choice and allow players to identify whether these factors are barriers to meeting nutrition goals. Establish whether players feel their dietary habits change depending on the season, i.e., pre- and competition season. Explores the impact of training on players′ food choice and dietary habits.
	Can you think of any other factors that might influence your intake throughout the week?	Provides insight into other factors that influence dietary habits, that may not have been highlighted by research previously.
Section two Current nutrition practices surrounding body composition assessment	Do you currently have any body composition goals? Do you have body fat percentage goals or lean muscle mass goals?	Establishes whether players have body composition goals and identifies whether goals are body fat or lean muscle mass.
	In the weeks leading into a DXA scan, can you describe changes (if any) to your dietary intake? Is this different to a normal training week when DXA scans are not taking place? In the days leading up to a DXA scan, can you describe changes (if any) to your dietary intake? Is this different to a normal training week when DXA scans are not taking place? Are there any foods you do or do not eat before a DXA scan? Do you feel any pressures leading into a DXA scan? Do you think these pressures (if any) influence your intake?	Aims to identify if players change their dietary intake in the weeks leading into a body composition assessment period and provides insight into the types of dietary habits that occur. Aims to identify if players change their dietary intake in the days leading up to a body composition assessment period and provides insight into the types of dietary habits that occur. Explores players’ feelings surrounding body composition assessment periods and perceptions body composition and goals.
Section Three Nutrition education and the role of the sports dietitian in team sport	Moving forward, is there any information regarding nutrition you are unsure about? What types of education would help you? (i.e., one on one consultations, group education, cooking classes?)	Provides a platform for players to provide feedback on nutrition support and education required to support dietary intake meeting nutrition goals. Identifies types of education players prefer and provides an insight into preferred learning styles.

Abbreviations: DXA: Dual Energy X-ray Absorptiometry.

**Table 2:** A summary of categories, sub-categories and themes identified from semi-structured interviews.

Research Question	Categories	Sub-Categories	Themes
What factors influence food choice and dietary behaviors of AF players?	1. Body composition	1.1. Perceived pressures associated with body composition assessment and goals 1.2. Dietary strategies used to meet body composition goals 1.3. Experienced AF players felt less pressure meeting body composition goals	Players feel pressure from peers, high performance teams, S&C, and coach to meet body composition goals. Players modify dietary intake leading up to DXA by restricting carbohydrate and/or discretionary foods and manipulating fluid intake. Experienced players feel less pressure to achieve body composition goals and focus more on performance.
	2. Interpersonal factors (i.e., peers, family, and access)	2.1. The influence of peers and family 2.2. The influence of mood on food choice	When players felt supported by their relationships in making healthy food choices, they were more likely to make positive food choices related to health. Players that felt decreased mood levels had a lack of motivation to make positive food choices.
	3. Stage of competition season (i.e., in season versus preseason)	3.1. Preseason influenced dietary intake (i.e., reduced carbohydrate intake) 3.2. Competition season influenced dietary intake (i.e., increased carbohydrate intake)	Players felt more pressure to meet body composition goals during preseason and reduced intake of carbohydrate surrounding DXA scans. A lack of time on main training days negatively influenced dietary intake. Players reported a greater intake of carbohydrates during competition season. Players experienced suppressed appetite post competition matches.
	4. Nutrition knowledge and support	4.1. Nutrition education and support preferred by AF players	Players prefer individual dietetic consultations to group presentations. Players feel they have good nutrition knowledge and are not seeking specific nutritional information.

Abbreviations: S&C: Strength and Conditioning, DXA: Dual Energy X-ray Absorptiometry, AF: Australian Football.

## Data Availability

Authors declare that the data supporting the findings of this study are available within the article. Due to the nature of this research and privacy of participants, additional supporting data is not available.
